# There is no F in APC: Using physiological fluoride-free solutions for high throughput automated patch clamp experiments

**DOI:** 10.3389/fnmol.2022.982316

**Published:** 2022-08-22

**Authors:** Markus Rapedius, Alison Obergrussberger, Edward S. A. Humphries, Stephanie Scholz, Ilka Rinke-Weiss, Tom A. Goetze, Nina Brinkwirth, Maria Giustina Rotordam, Tim Strassmaier, Aaron Randolph, Søren Friis, Aiste Liutkute, Fitzwilliam Seibertz, Niels Voigt, Niels Fertig

**Affiliations:** ^1^Nanion Technologies GmbH, Munich, Germany; ^2^Discovery Sciences, BioPharmaceuticals R&D, AstraZeneca, Cambridge, United Kingdom; ^3^Nanion Technologies Inc., Livingston, NJ, United States; ^4^Institute of Pharmacology and Toxicology, University Medical Center Göttingen, Georg-August University, Göttingen, Germany; ^5^German Center for Cardiovascular Research, Partner Site Göttingen, Göttingen, Germany; ^6^Cluster of Excellence “Multiscale Bioimaging: From Molecular Machines to Networks of Excitable Cells”, University of Göttingen, Göttingen, Germany

**Keywords:** automated patch clamp, ion channels, hERG, Na_V_1.5, physiological solutions, K_Ca_3.1, Na_V_1.7, fluoride

## Abstract

Fluoride has been used in the internal recording solution for manual and automated patch clamp experiments for decades because it helps to improve the seal resistance and promotes longer lasting recordings. In manual patch clamp, fluoride has been used to record voltage-gated Na (Na_V_) channels where seal resistance and access resistance are critical for good voltage control. In automated patch clamp, suction is applied from underneath the patch clamp chip to attract a cell to the hole and obtain a good seal. Since the patch clamp aperture cannot be moved to improve the seal like the patch clamp pipette in manual patch clamp, automated patch clamp manufacturers use internal fluoride to improve the success rate for obtaining GΩ seals. However, internal fluoride can affect voltage-dependence of activation and inactivation, as well as affecting internal second messenger systems and therefore, it is desirable to have the option to perform experiments using physiological, fluoride-free internal solution. We have developed an approach for high throughput fluoride-free recordings on a 384-well based automated patch clamp system with success rates >40% for GΩ seals. We demonstrate this method using hERG expressed in HEK cells, as well as Na_V_1.5, Na_V_1.7, and K_Ca_3.1 expressed in CHO cells. We describe the advantages and disadvantages of using fluoride and provide examples of where fluoride can be used, where caution should be exerted and where fluoride-free solutions provide an advantage over fluoride-containing solutions.

## Introduction

Automated patch clamp (APC) instruments are used for a wide variety of applications ranging from basic research into channelopathies and biophysical characteristics of ion channels, through to routine cardiac safety testing (Obergrussberger et al., 2021, 2022). Their use in cardiac safety screening has increased over the years and APC is now an established and accepted technique in most, if not all, safety testing laboratories. It is well-known that fluoride is often used in the internal solution in APC experiments to improve the seal resistance (Zeng et al., 2008) when external calcium (or other divalent cation) are present (Milligan et al., 2009; Obergrussberger et al., 2014, 2018). The mechanism is thought to involve the formation of CaF_2_ crystals at the interface between the pipette or micro-pore and the cell as described in a recent patent application (Lojkner et al., 2019). Even in manual patch clamp experiments, fluoride has been used to record Na_V_ channels for over 20 years (Cummins et al., 1999; Renganathan et al., 2000; Rugiero et al., 2003), despite known shifts in activation and steady-state inactivation curves of Na_V_1.9 (Rugiero et al., 2003; Coste et al., 2004), effects on the voltage dependence of conductance and steady-state inactivation of Na_V_1.7 (Jarecki et al., 2008), shifts in activation and inactivation of Na_V_1.3 (Meadows et al., 2002), and effects on persistent current of Na_V_1.3 (Chen et al., 2000). These effects on biophysical properties are not limited to Na_V_ channels, high concentrations of fluoride have been shown to increase mean open duration, and thereby open probability of L-type calcium channels (Ono and Arita, 1999), and influences current amplitude and inactivation of K_V_1.3 and K_2P_ channels (Herrmann et al., 2020). In addition to effects on biophysical properties of ion channels, fluoride binds to calcium making its use in experiments involving activation of ion channels by internal free calcium somewhat limited, and itself activates certain ion channels such as CFTR (Berger et al., 1998). It is also well-known that fluoride activates G-proteins when complexed with Al^3+^ present in trace amounts (Li, 2003) and inhibits protein phosphatase (Khatra and Soderling, 1978). Fluoride is used because it improves the seal and allows stable measurements to be performed over long periods of time (Zeng et al., 2008; Föhr et al., 2021). Alternative internal solutions which do not contain fluoride for recording CFTR (Billet et al., 2017; Froux et al., 2020; Becq et al., 2021) or TMEM16A (Obergrussberger et al., 2018) have been used successfully on automated patch clamp devices, but the solutions were not always physiological.

In most experiments run on automated patch clamp instruments, the use of fluoride in the internal solution is no problem, particularly when like is compared with like, i.e., activation and inactivation plots of Na_V_ channels are always run using internal fluoride. However, there may be some cases where fluoride-free, physiological solutions are required, for example, when comparing data with historical data which was recorded in fluoride-free solutions. Alternatively, it may be the case that fluoride cannot be used because of activation of ion channels, inhibition of phosphatase or binding of calcium ions. For these reasons, we have developed a method for high throughput APC experiments using the SyncroPatch 384 which allow fluoride-free, physiological solutions to be used with good success rates for GΩ and sub-GΩ seals. We demonstrate this using the ion channels Na_V_1.5 and Na_V_1.7 expressed in CHO (Charles River), hERG expressed in HEK293 (SB Drug Discovery), and K_Ca_3.1 (SK4) also expressed in CHO cells (Charles River). Additionally, preliminary experiments using human induced pluripotent stem cell-derived cardiomyocytes (hiPSC-CMs) were also performed.

## Materials and methods

### Cell culture and harvesting

The cell lines used here (hERG stably expressed in HEK 293 cells from SB Drug Discovery, Glasgow, United Kingdom, hNa_V_1.5; hNa_V_1.7, and K_Ca_3.1 stably expressed in CHO cells all from Charles River, United States) were cultured as previously described (Brueggemann et al., 2004; Brüggemann et al., 2008; Becker et al., 2013; Obergrussberger et al., 2014). In brief, these cells were cultured in T175 culture flasks in the media recommended by the supplier and passaged every 2–3 days when they were 50–80% confluent. The cells were passaged regularly to ensure that the cells were single when passaged and harvested. The cells were prohibited from reaching 100% confluency so that they remained healthy, single, and expressed the ion channel of interest when they were harvested into a cell suspension for recordings. hiPSC-derived cardiomyocytes were generated and cultured as previously described (Seibertz et al., 2022). Briefly, hiPSC cell line UMGi014-C.14 was derived from dermal fibroblasts of healthy male donor and the reprogramming to iPSCs was achieved using integration-free CytoTune iPS 2.0 Sendai Reprogramming Kit (Thermo Fisher Scientific) with the reprogramming factors OCT4, KLF4, SOX2, c-MYC. Directed differentiation into ventricular iPSC-CMs was performed *via* WNT signaling modulation and subsequent metabolic selection, as previously described (Kleinsorge and Cyganek, 2020). hiPSC-CMs were cultured at 37°C in 5% CO_2_ in a RPMI Medium 1640 (1X) + GlutaMax™ –I [+] 25 mM HEPES (Thermo Fisher Scientific) supplemented with B-27 (Thermo Fisher Scientific). Experiments were performed on hiPSC-CMs at day 41 post differentiation. All protocols were approved by the Ethics Committee of the University Medical Center Göttingen (No. 10/9/15 and 15/2/20). For experiments on the SyncroPatch 384, cells were harvested into suspension and suction was used to attract a cell to the patch clamp aperture of each well. Since it was a blind method for capture, cells had to be single with few clusters and the cell suspension was free from cell debris, as the presence of cell clusters and debris can decrease the success rate. Cell harvesting was performed as described previously (Brueggemann et al., 2004; Brüggemann et al., 2008; Becker et al., 2013; Obergrussberger et al., 2014) using TrypLE, other suitable enzymes, or even enzyme-free detachment protocols. Cells were then resuspended in extracellular recording solution at a density of ∼500,000 cells/ml.

### Automated patch clamp electrophysiology

All cells were recorded in the whole cell mode of the patch clamp technique using the SyncroPatch 384 (Nanion Technologies, Munich, Germany). Electrophysiological protocols were constructed, and data digitized using PatchControl 384 (Nanion Technologies, Munich, Germany). Cells were recorded on both single-hole NPC-384 or NPC-384FF chips with medium or high resistance (4–5.5 or 5–7 MΩ, respectively). In a subset of experiments involving K_Ca_3.1 multi-hole chips with 4 holes (NPC-384/NPC-384FF) 4X high resistance (2.2–3.2 MΩ) per well were used. Cells were harvested into suspension and added to the CellHotel of the SyncroPatch 384 where they were kept at 10°C, shaking at 200 rpm, until used for the experiment. The NPC-384 or NPC-384FF chips were filled with internal solution as indicated in Table 1 and ChipFill external solution. After cells were captured (indicated by an increase in R_Seal_ > 10 MΩ), a brief, transient addition of external solution containing 6 mM Ca^2+^ was made when standard internal solution and NPC-384 chips were used which was then exchanged to external recording solution before commencement of recordings. When fluoride-free internal solution and NPC-384FF chips were used, the ChipFill external solution was replaced by external recording solution (Table 1) after cells were captured.

**TABLE 1:** Composition of recording solutions for fluoride-containing (referred to as standard internal) and fluoride-free (referred to as FF internal) experiments.

Constituent	Internal recording solution			External recording solution	ChipFill external solution (divalent-free)
	Standard	Standard-K_Ca_3.1*	FF	Standard (incl. K_Ca_3.1)/FF	Standard (incl. K_Ca_3.1)/FF
K-gluconate		30	120		
K-Fluoride	120	80			
NaCl	10	10	10	80	140
NMDG-Cl				60	
KCl	10	10	10	4	4
CaCl_2_				2	
MgCl_2_				1	
EGTA	5	10	5		
Glucose				5	5
HEPES	10	10	10	10	10
pH	7.2	7.2	7.2	7.4	7.4
Osmolarity (mOsm)	∼285	∼285	∼285	∼289	∼289

Concentrations are given in mM. Note that for experiments involving K_Ca_3.1, CaCl_2_ was added to the internal solution as described in Section “Automated patch clamp electrophysiology.”

*In recordings with free [Ca^2+^ > 1 μM] 10 mM HEDTA was used instead of EGTA.

hERG currents were recorded using a double step voltage protocol from a holding potential of −80 mV to +60 mV for 500 ms followed by a step to −40 mV for 500 ms repeated every 15 s. Peak amplitude was measured at the start of the second voltage step (I_Tail_). Current-voltage relationships were recorded using the same voltage-step to +60 mV, but then followed by voltage steps from −120 to 60 mV with 10 mV increments.

Na_V_1.5 currents were measured using a 3-step protocol, the first step from −120 to 0 mV, the second step from −100 to 0 mV and the 3rd step from −80 to 0 mV, repeated every 10 s. Current-voltage (IV) relationships were recorded using the voltage-step protocol: For the activation IV, a step pulse of 30 ms was applied, starting from −70 mV and increasing in 10 mV increments up to 40 mV with every sweep, at a sweep interval of 2 s. For analysis, the current response to each step was normalized to the cell’s peak current before averaging across all cells. Holding potential was −120 mV.

The inactivation IV pulse protocol consisted of a 500 ms pre-pulse, starting from −100 mV and increasing in 5 mV increments with every sweep up to 20 mV, followed by a 50 ms depolarizing step to 0 mV, at a sweep interval of 5 s. For analysis, each of the current responses to the 50 ms step to 0 mV was normalized to the current response to the 0 mV step following the 500 ms pre-pulse at −120 mV before averaging across all cells. Holding potential was −120 mV.

K_Ca_3.1 was recorded using a voltage ramp protocol from −120 to +60 mV over 198 ms, intersweep holding potential was −80 mV and sweep interval was 5 s. K_Ca_3.1 was activated by internal perfusion of free calcium at 0.3, 1, or 3 μM calculated using Cabuf (freeware from KU Leuven, Belgium).

Ca_V_ currents from hiPSC-CMs were recorded using a voltage ramp-step protocol, with a ramp from −80 to −40 mV over 300 ms followed by a step from −40 to 0 mV for 100 ms, holding potential was −80 mV. The ramp section of the protocol was used to inactivate Na_V_ currents. Only the current response to the step to 0 mV is shown in the figure for hiPSC-CMs.

Each well of the SyncroPatch 384 has an individual headstage of the amplifier and, therefore, each well is denoted as *n* = 1. For success rate and IC_50_ values across plates, n represents results from 1 NPC-384 chip. With PatchControl 384, parameters such as seal resistance, capacitance and series resistance were determined from each well after application of a test pulse. All parameters are monitored over time and can be recorded for individual experiments.

#### Method of using fluoride-free physiological solutions on the SyncroPatch 384

To allow for fluoride-free recordings, a combinatory approach is adopted on the SyncroPatch 384 consisting of the usage of a specialized, fluoride-free consumable called NPC-384FF in combination with a proprietary, fully automated method for preparing the NPC-384FF prior to execution of the fluoride-free recording. The automated method of NPC-384FF preparation is available on all SyncroPatch 384 devices.

#### Quality control parameters

Cells had to pass quality control (QC) parameters to be included in the analysis. For some experiments R_Seal_ > 1 GΩ whereas for other experiments R_Seal_ > 0.25 GΩ was used for single- or equivalent in multi-hole chips. In addition to this, I_Peak_ > 150 pA for hERG; I_Peak_ < −100 pA for Na_V_1.5 or Na_V_1.7 and I_Peak_ > 350 pA for single-hole chips and I_Peak_ > 1000 pA for multi-hole chips (4X) for K_Ca_3.1 was also used. In order to guarantee appropriate voltage clamp in our comparisons, the analysis of IV-relationships for Na_V_1.5 and Na_V_1.7 recordings included additional quality control parameters on capacitance > 2 pF, current size as well as R_series_ that was constrained to −3.0 nA and 20 MΩ, respectively.

#### Automated patch clamp solutions

See Table 1.

### Data analysis

The SyncroPatch 384 platforms have a software package consisting of PatchControl 384 (for data acquisition) and DataControl 384 (for data analysis; both Nanion Technologies, Munich, Germany). Current-voltage plots were calculated using DataControl 384 and fit with Boltzmann equations: $I\,\left(V\right)=I\,m\,i\,n+\frac{I\,m\,a\,x-I\,m\,i\,n}{1+e^{-\frac{V-V\,h\,a\,l\,f}{S\,l\,o\,p\,e}}}$ for a sigmoidal fit for hERG IV and inactivation Na_V_1.5 and Na_V_1.7 IVs. In addition, an extended Boltzmann equation was used: $G\,\left(V\right)=\frac{I\,\left(V\right)}{V-E\,r\,e\,v}=\left(G\,m\,i\,n+\frac{G\,m\,a\,x-G\,m\,i\,n}{1+e^{-\frac{V-V\,h\,a\,l\,f}{S\,l\,o\,p\,e}}}\right)$ to obtain V_half_ values for activation protocols for Na_V_1.5 and Na_V_1.7 where I(V) is the amplitude of the current and G(V) the conductance at voltage V, Erev is the reversal potential. Vhalf is the membrane potential at half-maximal activation or inactivation and Slope is the slope factor.

Concentration response curves were calculated using either cumulative additions or a single point addition approach. When cumulative additions were done, all concentrations of compound were added to each well, in increasing concentrations, and concentration response curves were calculated individually for each well. When single point additions were done, a single concentration of compound was added to each well and the IC_50_ was calculated across multiple wells. IC_50_ analysis was done with DataControl 384, concentration response curves were fit with a Hill equation:

$$R\,e\,s\,p\,o\,n\,s\,e=M\,i\,n+\frac{M\,a\,x-M\,i\,n}{1+{\left(\frac{E\,C\,50}{Conc.}\right)}^{H\,i\,l\,l}}.$$

### Statistics

Data are presented as means ± S.D or means ± S.E.M. Differences between groups were tested using the Student’s *t*-test for normally distributed data. *P*-values are reported in the figures. N represents the number of wells examined or the number of chips as indicated in the figures and/or figure legends.

## Results

### High throughput automated patch clamp to assess effects of internal fluoride on success rate and seal resistance

In APC experiments, high success rates have been reported using internal solutions without fluoride for several targets, such as CFTR, with success rates for completed experiments > 80% (Billet et al., 2017; Brüggemann et al., 2017; Froux et al., 2020; Becq et al., 2021). However, when switching to more physiological solutions, such as K-chloride or K-gluconate-based internal solutions, success rates very rarely reach acceptable ranges for high throughput APC. In fact, under these conditions we observed 9.3 ± 6.1% (R_Seal_ > 1 GΩ + I_Tail_ > 150 pA) and 18.6 ± 6.3% (R_Seal_ > 0.25 GΩ + I_Tail_ > 150 pA) using hERG expressed in HEK cells (*n* = 16 NPC-384 chips), in agreement with success rates reported by Zeng et al. (2008). This has posed a limitation in the use of fluoride-free, physiological internal solutions for high throughput APC and was the basis for the development of a special type of consumable, the NPC-384FF, which we have used throughout this study to allow the usage of K-gluconate (or K-chloride) or fluoride-based internal solutions under the same experimental conditions.

**FIGURE 1:**
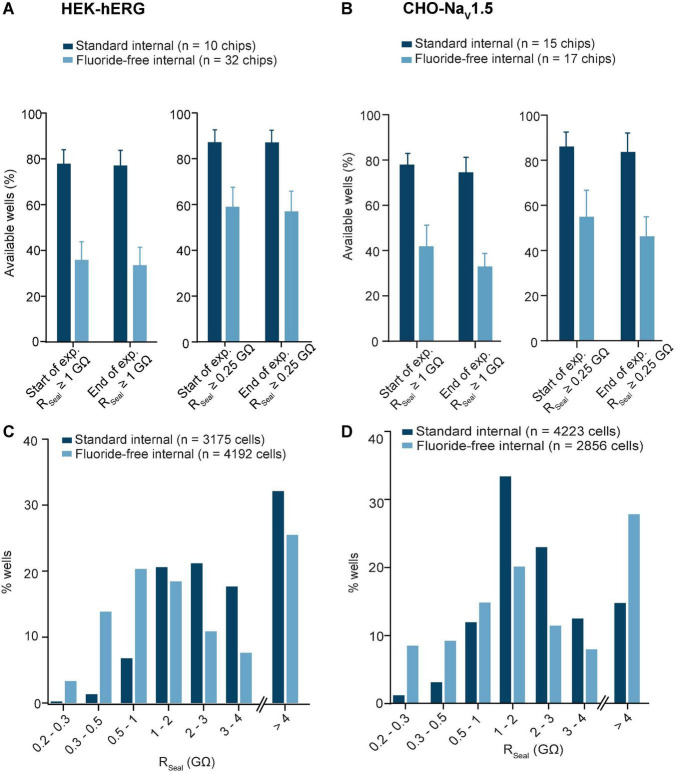
HEK and CHO cells recorded in standard and fluoride-free internal solution. **(A)** HEK cells expressing hERG were captured with almost 80% success rate for R_Seal_ ≥ 1 GΩ + I_Tail_ > 150 pA and almost 40% success rate for R_Seal_ ≥ 1 GΩ in fluoride-free internal solution (left). If a QC cutoff of R_Seal_ ≥ 0.25 GΩ + I_Tail_ > 150 pA was used, the success rate was increased in both conditions (right). **(B)** CHO cells expressing Na_V_1.5 were captured with almost 80% success rate for R_Seal_ ≥ 1 GΩ + I_Peak_ < –100 pA and over 40% success rate for R_Seal_ ≥ 1 GΩ in fluoride-free internal solution (left). If a QC cutoff of R_Seal_ ≥ 0.25 GΩ + I_Peak_ < –100 pA was used, the success rate was increased in both conditions (right). **(C)** Histogram plot of the R_Seal_ values for HEK-hERG cells in standard and fluoride-free internal solution. For R_Seal_ > 4 GΩ, although they are high giga-seals, the seal resistance is not accurately calculated and is therefore shown as an extra bar and these R_Seal_ values were not included in the calculation for R_Seal_ (Table 2). **(D)** Histogram plot of the R_Seal_ values for CHO-Na_V_1.5 cells in standard and fluoride-free internal. For R_Seal_ > 4 GΩ, although they are high giga-seals, the seal resistance is not accurately calculated and is therefore shown as an extra bar and these R_Seal_ values were not included in the calculation for R_Seal_ (Table 3).

First, we evaluated the success rates for seal formation for fluoride-free internal solution compared with standard, fluoride-containing, internal solution using two standard cell culture cell lines. HEK cells expressing hERG as wells as CHO cells expressing Na_V_1.5 channels were used on the SyncroPatch 384 using either NPC-384 chips and standard internal solution containing fluoride, or NPC-384FF chips with fluoride-free internal solution. For hERG, a classical double voltage-step protocol was used to record hERG tail currents and we determined the number of wells available for recording under both conditions. Using single-hole chips, HEK cells expressing hERG were recorded with a success rate of 77.9 ± 6.1% (*n* = 10 NPC-384 chips) for R_Seal_ > 1 GΩ + I_Tail_ > 150 pA (at the start of the experiment) when standard K-fluoride was used in the internal solution and 35.9 ± 7.9% (*n* = 32 NPC-384FF chips) when K-gluconate was used in the internal solution (Figure 1A). The success rate increased to 87.2 ± 5.4% (*n* = 10 NPC-384 chips) in K-fluoride and 59.0 ± 8.5% (*n* = 32 NPC-384FF chips) in K-gluconate when a QC cutoff for R_Seal_ > 0.25 GΩ + I_Tail_ > 150 pA was used. Despite the reduced number of available wells when fluoride-free solution was used, the sealing properties of the recorded cells only slightly differed in their R_Seal_ distribution pattern as illustrated in the histograms (Figure 1C), however, a small, but significant reduction for mean R_Seal_ values was observed (Table 2). Importantly, this did not have a large impact on the stability of the recording over time as only few cells were lost during the experiments and the success rate was similar at the end of the experiment (t = 15 min) as at the start of the experiment (Figure 1A). The differences in mean R_Seal_ might be due to the fact that for the fluoride-free approach, the formation of CaF_2_ crystals is absent and no elevated external Ca^2+^ levels are used throughout the entire recording resulting in a reduced success rate. Similar results were observed using CHO cells expressing Na_V_1.5 channels where a 1-step voltage protocol was used to elicit Na_V_1.5 peak currents. Again, we first determined the number of available wells in single-hole chips (i.e., the number that passed the QC parameters for R_Seal_ and I_Peak_) and for Na_V_1.5-CHO we observed a success rate of 78.0 ± 4.9% (*n* = 15 NPC-384 chips) for R_Seal_ > 1 GΩ + I_Peak_ < −100 pA (at the start of the experiment) when K-fluoride was used in the internal solution and 41.9 ± 8.6% (*n* = 17 NPC-384FF chips) when K-gluconate was used in the internal solution (Figure 1B). The success rate increased to 86.1 ± 6.4% (*n* = 15 NPC-384 chips) in K-fluoride and 55.0 ± 11.7% (*n* = 17 NPC-384FF chips) in K-gluconate when a QC cutoff R_Seal_ > 0.25 GΩ + I_Peak_ < −100 pA was used. In line with our results with hERG-HEK cells, we observed a similar R_Seal_ distribution pattern for both conditions as illustrated in the histogram (Figure 1D) and a significant difference in mean R_Seal_ values for CHO cells in standard and fluoride-free conditions (Table 3). Similar to hERG-HEK, success rates remained stable over the time course of the experiment, any differences can probably be also attributed to the absence of CaF_2_ crystal formation as described above. Taken together, the results from both standard cell culture cell lines (HEK293 or CHO) expressing either Na_V_1.5 or hERG channels suggest that the fluoride-free approach can be used as an alternative to fluoride-containing internal solution in automated patch clamp experiments with reduced, but acceptable success rates. Therefore, our aim was to minimize experimental variability (such as recording solutions, voltage protocols, recording temperature, expression system, and culturing conditions) and provide a systematic comparison of biophysical and pharmacological parameters for four different targets depending on the internal solutions used.

**TABLE 2:** Parameters for R_Seal_, I_Tail_, cell capacitance, R_Series_, Rundown, V_half_, and IC_50_ values for HEK cells expressing hERG for standard internal and fluoride-free internal solution.

Parameter	Standard internal	Fluoride-free internal
R_Seal_ (GΩ)	2.23 ± 0.02 (2155)	1.36 ± 0.02 (3123)*
I_Tail_ (nA)	0.70 ± 0.01 (3175)	0.99 ± 0.01 (4192)***
Capacitance (pF)	16.2 ± 0.3 (387)	15.9 ± 0.4 (313)
R_Series_ (MΩ)	8.6 ± 0.2 (379)	10.2 ± 0.2 (317)***
Rundown over 18 min (%/min)	0.10 ± 0.05 (58)	0.07 ± 0.07 (42)
V_half_ (mV)	−93.0 ± 0.5 (190)	−92.1 ± 2.3 (261)
IC_50_ Terfenadine (nM)	237 ± 26 (52)	337 ± 53 (17)
IC_50_ Verapamil (nM)	394 ± 17 (54)	457 ± 37 (29)

R_Seal_ values > 4 GΩ could not be accurately calculated, therefore only R_Seal_ ≤ 4 GΩ were used for the calculation of the mean. Shown are mean ± S.E.M, number of cells shown in parentheses.

*P < 0.05, ***P < 0.001, unpaired Student’s t-test.

**TABLE 3:** Parameters for R_Seal_, I_Peak_, rundown, V_half_ Activation, V_half_ Inactivation, and tetracaine IC_50_ for CHO cells expressing Na_V_1.5 for standard internal and fluoride-free internal solution.

Parameter	Standard internal	Fluoride-free internal
R_Seal_ (GΩ)	1.89 ± 0.02 (3598)	1.39 ± 0.02 (2061)***
I_Peak_ (nA)	−1.56 ± 0.01 (4537)	−1.53 ± 0.01 (3336)
Capacitance (pF)	13.9 ± 0.2 (1043)	13.8 ± 0.4 (491)
R_Series_ (MΩ)	7.6 ± 0.1 (1043)	9.8 ± 0.1 (491)***
Rundown over 12 min (%/min; V_hold_ −120 mV)	0.5 ± 0.1 (133)	0.4 ± 0.1 (52)
V_half_ Activation (mV)	−37.5 ± 0.4 (484)	−25.2 ± 0.5 (241)***
V_half_ Inactivation (mV)	−68.4 ± 0.4 (728)	−53.6 ± 0.6 (165)***
IC_50_ Tetracaine (μM, V_hold_ −120 mV)	35.6 ± 9.4 (4 chips)	41.8 ± 9.3 (3 chips)

R_Seal_ values > 4 GΩ could not be accurately calculated, therefore only R_Seal_ ≤ 4 GΩ were used for the calculation. Shown are mean ± S.E.M, number of cells shown in parentheses. For tetracaine concentration response curves, single point additions were made and the concentration response curve calculated across the whole chip. Shown are mean ± S.E.M for the number of chips given in parentheses.

***P < 0.001, unpaired Student’s t-test.

### Effect of internal fluoride on currents, biophysical properties, and pharmacology of hERG expressed in HEK cells

**FIGURE 2:**
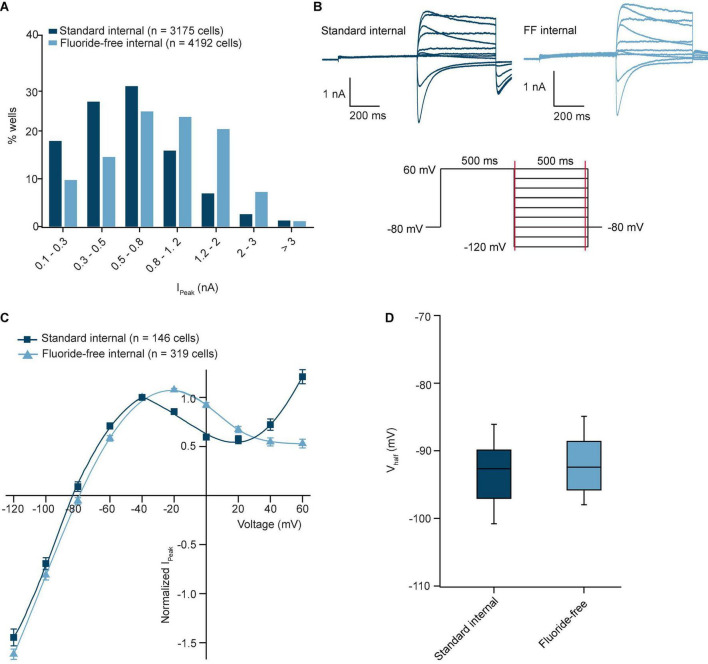
hERG currents in standard internal and fluoride-free internal solution. **(A)** Histogram plot of I_Peak_ values for standard internal and fluoride-free internal. There was a tendency to higher peak amplitudes in fluoride-free internal solution vs. standard internal. **(B)** Current-voltage traces of hERG using standard internal (left) and fluoride-free internal (right). The voltage protocol is shown underneath. **(C)** IV curves for an average of 146 cells in standard internal and 319 cells in fluoride-free solution are shown overlaid. A standard Boltzmann equation (see Section “Data analysis”) was used to fit the curve from –120 to –20 mV. **(D)** V_half_ values for hERG recorded in standard internal and fluoride-free internal. The V_half_ values of 190 cells in standard internal and 261 cells in fluoride-free internal were not significantly different using a Student’s *t*-test (*P* > 0.05).

From the hERG-HEK recordings shown in Figures 1A,C we also extracted peak current levels from available wells as displayed in the histogram (Figure 2A). Interestingly, the data showed a difference in the distribution pattern in line with a significantly higher mean peak current level when using fluoride-free solution (Table 2) and single-hole chips. To further understand this difference, we examined the IV relationship using a classical double step inactivation protocol with increasing voltages after initial depolarization to +60 mV using standard and fluoride-free internal solution (Figure 2B) and found a similar IV-relationship that started to deviate for voltages more positive to −40 mV (Figure 2C). The currents were therefore normalized to −40 mV and fitted with a standard Boltzmann equation and resulted in V_half_ values of −93.0 ± 0.5 mV (*n* = 190 cells) for standard and −92.1 ± 2.3 mV (*n* = 201 cells) for fluoride-free internal solution with the data pools found to be not significantly different (Figures 2C,D and Table 2). The results imply that voltage dependence per se does not account for the difference in peak current level observed when fluoride-free solution was used. We can also rule out differences in cell size or access resistance as capacitance values were the same in both conditions and R_series_ values were only slightly (but significantly) different (Table 2). Furthermore, the recordings were always performed in a comparable way on the same day using the same cells, recording temperature, external solution, and voltage protocol, with the only difference being the internal solution (standard or fluoride-free). This excludes any errors that may have arisen due to different recording conditions. Therefore, the reasons for the differences in increased currents in fluoride-free solution is unclear and further studies, such as detailed IV properties and a kinetic analysis, are required to elucidate the underlying reasons.

**FIGURE 3:**
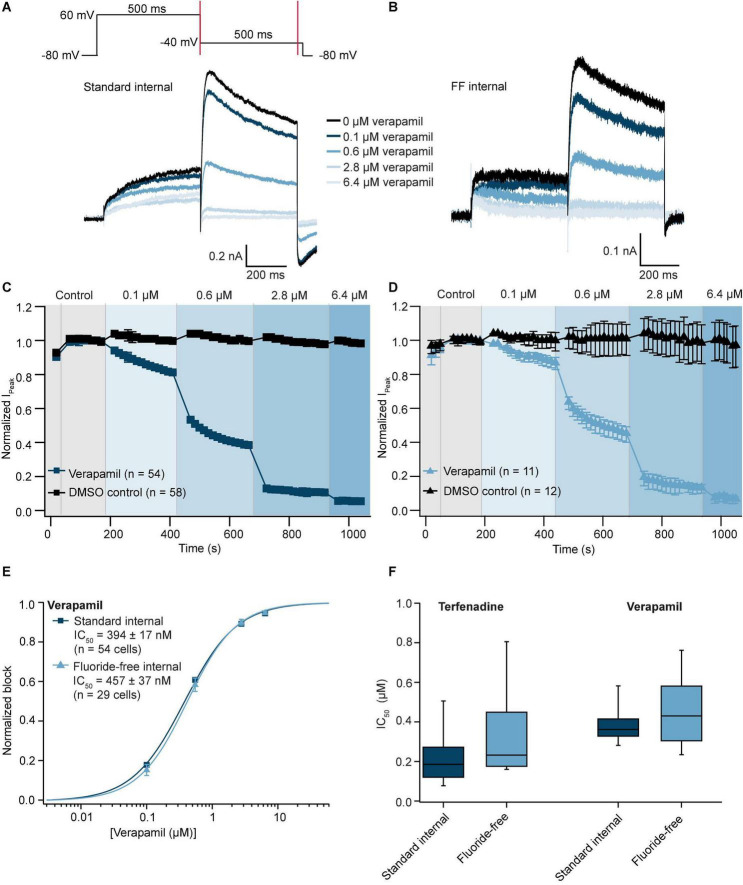
Pharmacology of hERG recorded in standard and fluoride-free internal solution. **(A,B)** Raw current traces from an example HEK cell expressing hERG recorded in standard internal **(A)** and fluoride-free internal **(B)** solution was blocked by increasing concentrations of verapamil [voltage protocol is shown at the top of panel **(A)**]. **(C)** Corresponding time course of block by verapamil in standard internal **(C)** and fluoride-free internal **(D)**, 54 or 11 cells, respectively. Time course of 58/12 cells in DMSO control is also shown within panels **(C,D)**. **(E)** Concentration response curve for verapamil in standard internal solution or fluoride-free solution are shown overlaid. **(F)** IC_50_s for terfenadine and verapamil recorded using standard internal or fluoride-free internal solution are shown as a box plot. The IC_50_s for verapamil and terfenadine recorded in standard internal or fluoride-free internal solution were not significantly different using a Student’s *t*-test (*P* > 0.05).

To investigate the influence of internal fluoride on the pharmacology of hERG channels we compared the IC_50_s of verapamil and terfenadine in the presence and absence of internal fluoride. Figures 3A,B show traces from an example cell in the presence of increasing concentrations of verapamil in standard and fluoride-free internal solution, respectively. Figures 3C,D show the time course of the experiment for I_Tail_ current (at −40 mV) plotted over time using either standard (Figure 3C) or fluoride-free internal solution (Figure 3D) for an average of 54 and 11 cells, respectively. Also shown in Figures 3C,D is the vehicle control (0.3% DMSO, *n* = 58 cells for standard internal solution and 12 cells for fluoride-free internal) where rundown was low for both groups, <2% over 18 min (Table 2) indicating I_Tail_ current amplitude was stable regardless of internal solution used (*P* = 0.7, Student’s *t*-test). hERG was blocked by verapamil in a concentration-dependent manner with similar IC_50_s and the concentration-response curves overlaid almost exactly (Figure 3E, for terfenadine, see Supplementary Figure 1). The spread of IC_50_s for terfenadine and verapamil was higher when fluoride-free internal solution was used (Figure 3F) but the IC_50_s were not different to those recorded in standard internal solution (Figure 3F and Table 2). Taken together, despite differences in tail current amplitude, hERG channel voltage dependence of inactivation, recording stability (rundown) as well as pharmacology for the compounds tested seem independent of whether fluoride-free or standard internal solution was used, which has also been suggested by other studies (Zeng et al., 2008) implying that the effect of internal fluoride might not be generalized per se and rather depends on subtype specific modulation of ion channel activity.

### Effect of internal fluoride on seal resistance, stability, biophysics, and pharmacology of Na_V_1.5 expressed in CHO cells

**FIGURE 4:**
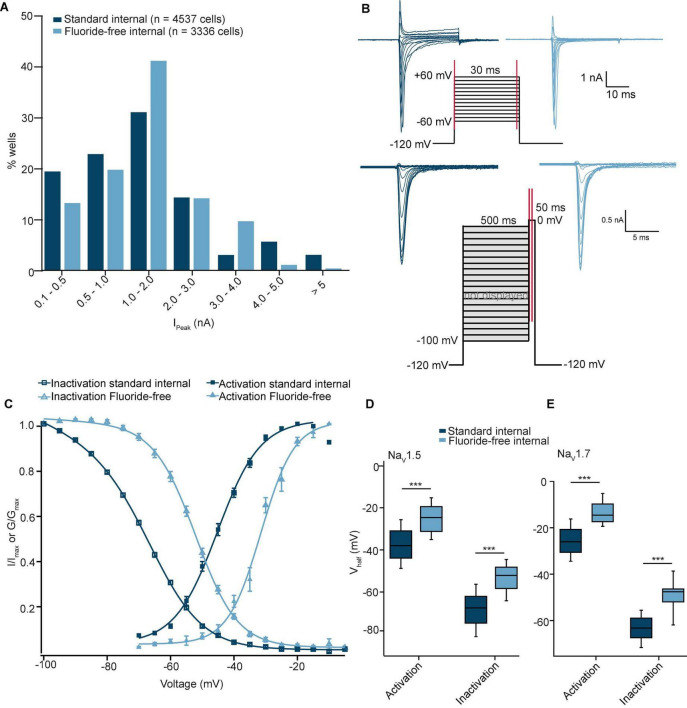
Na_V_1.5 recorded in standard and fluoride-free internal solution. **(A)** Histogram plot of I_Peak_ values recorded using standard internal and fluoride-free internal solution. **(B)** Current-voltage traces of Na_V_1.5 expressed in CHO cells using an activation protocol (top) and inactivation protocol (bottom) for both conditions. **(C)** Activation (fit with an extended Boltzmann equation, see Section “Data analysis”) and inactivation IV curves (fit with a Boltzmann equation, see Section “Data analysis”) for Na_V_1.5 in standard internal and fluoride-free solution are shown overlaid. **(D)** The V_half_ values of activation of 484 cells in standard internal were significantly more negative compared with 241 cells in fluoride-free internal. The same shift to more negative V_half_ of inactivation was also found for 728 in standard internal fluoride compared with 165 cells in fluoride-free internal (Student’s *t*-test, ***P < 0.001). **(E)** A shift in the V_half_ values of activation and inactivation was also observed for Na_V_1.7 where V_half_ values for activation of 433 cells in standard internal were significantly more negative compared with 29 cells in fluoride-free internal and the V_half_ of inactivation for 493 in standard internal fluoride were significantly more negative compared with 14 cells recorded in fluoride-free internal solution (Student’s *t*-test, ***P < 0.001).

From the experiments shown in Figures 1B,D, the peak current levels from available wells are displayed in Figure 4A as a histogram. In contrast to experiments involving hERG, the data showed a very similar distribution pattern consistent with a mean peak current amplitude that was not significantly different between the two groups (*P* = 0.2, Student’s *t*-test; Table 3). Furthermore, currents were stable during the time course of the experiment for both groups, with current rundown < 2% over ∼12 min (Table 3). Next, we investigated the effect of internal fluoride on the voltage dependence of activation and fast inactivation. Looking at the literature, there is a relatively large variability in V_half_ values reported for Na_V_1.5 due to different experimental parameters including voltage protocol, recording solutions, co-expression with β-subunits, recording temperature, holding potential, or cell expression system, the values range from −25 mV (Liu et al., 2003) to −50 mV (McNulty and Hanck, 2004). For this reason, we kept all experimental parameters the same, and only changed the internal solution in order to examine only the effect of fluoride on the V_half_ of activation and inactivation of Na_V_1.5. Figure 4B shows the voltage protocols used for activation and inactivation of Na_V_1.5 channels with example traces for both conditions. The results of these experiments are illustrated in Figures 4C,D where the V_half_ of activation was −25.2 ± 0.5 mV (*n* = 241) for fluoride-free and -37.5 ± 0.4 mV (*n* = 484) for standard internal solution. A similar difference was observed for the V_half_ of inactivation with −53.6 ± 0.6 mV (*n* = 165) in fluoride-free and −68.4 ± 0.4 mV (*n* = 728) using standard internal solution. There is a clear hyperpolarizing effect of fluoride on the voltage sensitivity of Na_V_1.5 channels which is highlighted in the boxplots shown in Figure 4D with ∼12 mV difference for V_half_ of activation and ∼15 mV for V_half_ of inactivation. Using the same voltage protocols as highlighted in Figure 4B we observed a similar hyperpolarizing shift for voltage activation/inactivation of Na_V_1.7 expressed in CHO cells when standard K-fluoride internal solution was used. This suggests a common phenotype for the shift of voltage activation/inactivation in Na_V_1.5 and Na_V_1.7 channels, however, further studies would be required to confirm whether other members of the Na_V_ family are affected in a same manner. In fact, a previous report indicates sub-type specific differences associated with the use of internal fluoride solutions (Coste et al., 2004).

**FIGURE 5:**
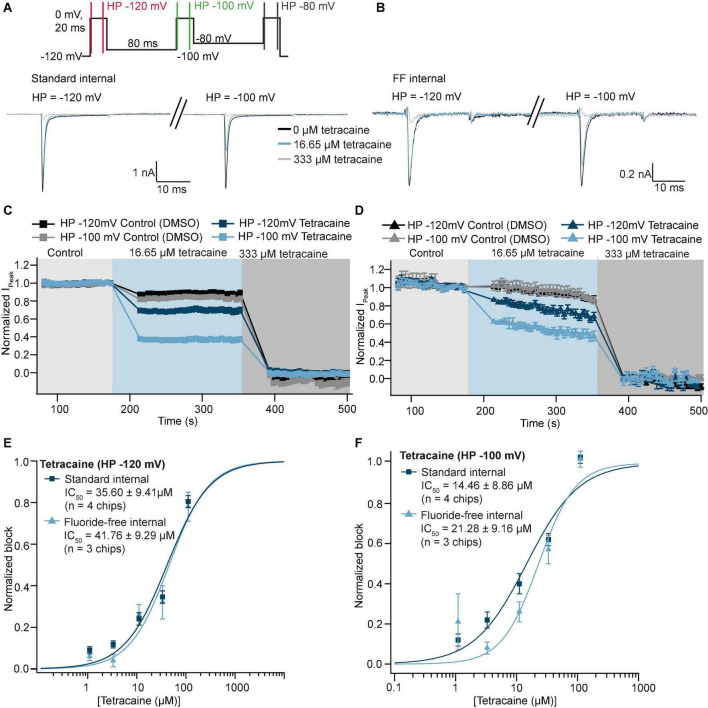
Pharmacology of Na_V_1.5 recorded in standard and fluoride-free internal solution. **(A)** Raw current traces from an example CHO cell expressing Na_V_1.5 recorded in standard internal solution were blocked by tetracaine using a 3-step protocol (voltage protocol shown at the top). Traces elicited by the first (holding potential –120 mV) and second (holding potential –100 mV) step are shown. **(B)** Raw current traces from an example CHO cell expressing Na_V_1.5 recorded in fluoride-free internal solution were blocked by tetracaine. **(C)** Corresponding time course of block by tetracaine in standard internal solution for an average of 23 wells (at different holding potentials), time course in DMSO for an average of 41 wells is also shown. **(D)** Corresponding time course of block by tetracaine in fluoride-free internal solution for an average of 10 wells (at different holding potentials), time course in DMSO for an average of 35 wells is also shown. **(E)** Concentration response curves for tetracaine in standard internal solution or fluoride-free solution at a holding potential of –120 mV are shown overlaid. **(F)** Concentration response curves for tetracaine in standard internal solution or fluoride-free solution at a holding potential of –100 mV are shown overlaid.

To test whether fluoride influences pharmacology of Na_V_1.5 we used the known Na_V_ channel blocker, tetracaine. This was applied as a single concentration of compound to each well, followed by a full block concentration (333 μM). Using a 3-Pulse protocol we were able to compare the block of Na_V_1.5-mediated currents by tetracaine at three different holding potentials as shown in Figures 5A,B (example traces are shown for the first two pulses only, corresponding to a holding potential of −120 and −100 mV). Figures 5C,D shows the peak current at 0 mV over time using two different holding potentials and tetracaine concentrations as well as vehicle only (0.3% DMSO) applied from the extracellular side. As expected, the potency of inhibition increased with a more depolarized holding potential for both conditions consistent with local anesthetics preferably binding to the inactivated states (Hille, 1977; Hondeghem and Katzung, 1977; Bean et al., 1983; Li et al., 1999). Importantly, the concentration response curves for tetracaine overlaid almost exactly for individual voltages and were not statistically significantly different (Student’s *t*-test, *P* > 0.05) for 4 NPC-384 chips in standard internal and 3 NPC-384FF chips in fluoride-free internal solution. However, the IC_50_s for tetracaine under both conditions decreased ∼4–7 fold from holding potentials of −120 mV compared with −100 and −80 mV (Figures 5E,F and Supplementary Table 1) comparable with values reported for lidocaine in the literature (Bean et al., 1983; Rapedius et al., 2019). In this way, the IC_50_ of tetracaine on Na_V_1.5 seems independent of whether fluoride is present or not, which is somewhat surprising given the clear hyperpolarizing effect of internal fluoride on voltage activation/inactivation of Na_V_1.5 (and Na_V_1.7) and the dependence of tetracaine IC_50_ on holding potential. Further studies are needed to elucidate the molecular mechanisms of these differences and how they apply to other compounds.

### Using fluoride-free solution to record K_Ca_3.1 (SK4) channels in the presence of internal Ca^2+^

**FIGURE 6:**
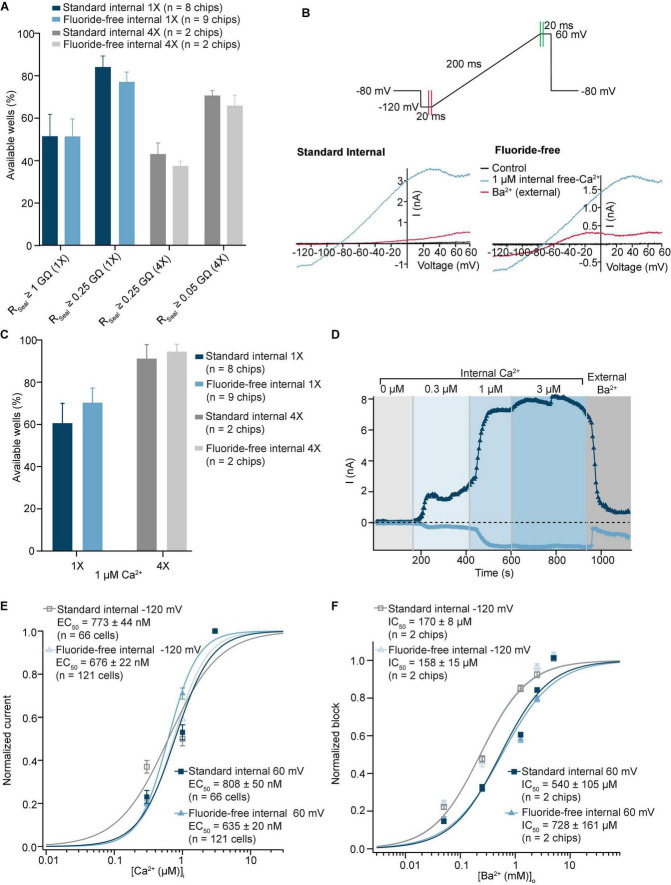
Activation by internal free Ca^2+^ of K_Ca_3.1 recorded in standard and fluoride-free internal solution. **(A)** CHO cells expressing K_Ca_3.1 were captured with almost 50% success rate for R_Seal_ ≥ 1 GΩ with standard and in fluoride-free internal solution (left). If a QC cutoff of R_Seal_ ≥ 0.25 GΩ was used, the success rate was increased in both conditions (right). Similar success rates were also achieved with multi-hole (4X) chips (gray). **(B)** Raw current traces from example CHO cell expressing K_Ca_3.1 recorded in both standard and fluoride-free internal solution, activated by 1 μM free internal Ca^2+^ and blocked by 1 mM Ba^2+^ (external). **(C)** Success rates after applying initial R_Seal_ (>0.25 GΩ) and current QC plot of I_Peak_ values at –120/60 mV for standard and fluoride-free solutions after internal application of 1 μM free Ca^2+^. **(D)** IT plot during application of internal free Ca^2+^ with 0.3, 1, and 3 μM free internal Ca^2+^ and subsequent block by external Ba^2+^ (1 mM) at –120/60 mV. **(E)** Current values at –120 or 60 mV in increasing concentrations of internal free Ca^2+^ were normalized to maximum peak amplitude and fit using a Hill equation which shows a slightly increased EC_50_ in standard internal solutions for both voltages. Shown are mean ± S.E.M. **(F)** Current values at –120 or 60 mV in increasing concentrations of external Ba^2+^ were normalized to maximum peak amplitude and fit using a Hill equation which showed no difference in the IC_50_ at –120 mV between standard internal (170 ± 8 μM, *n* = 2 chips) and fluoride-free internal (158 ± 15 μM, *n* = 2 chips) or 60 mV, although the IC_50_ was higher at 60 vs. –120 mV for both groups (540 ± 105 μM, *n* = 2 chips in standard external and 728 ± 161 μM, *n* = 2 chips in fluoride-free internal solution). IC_50_ values are given as mean of 2 chips ± S.D.

We also tested the effect of internal fluoride for an ion channel activated by internal Ca^2+^ and used the internal perfusion of free Ca^2+^ to activate K_Ca_3.1 (SK4) channels. CHO cells expressing K_Ca_3.1 were captured with almost 50% success rate for R_Seal_ ≥ 1 GΩ with standard and fluoride-free internal solution (Figure 6A), if a QC cutoff of R_Seal_ ≥ 0.25 GΩ was used, the success rate was increased in both conditions (Figure 6A). As automated patch clamp recordings are sometimes performed using multi-hole chips to improve success of experiments, we also used multi-hole NPC-384 and NPC-384FF chips with 4 holes per well (4X). The success rates for cell capture and sealing were similar using 4X chips compared with single hole (1X) chips for both standard internal and fluoride-free internal solution (Figure 6A). Note that when 4X chips were used, R_Seal_ > 0.25 GΩ is equivalent to R_Seal_ > 1 GΩ with 1X chips and R_Seal_ > 0.05 GΩ is equivalent to R_Seal_ > 0.25 GΩ with 1X chips. A ramp voltage protocol from −120 to 60 mV was applied from a holding potential of −80 mV (Figure 6B). After exchange of the intracellular solution from 0 to 1 μM free intracellular Ca^2+^ an outwardly rectifying current was observed that was inhibited by external application of 1 mM Ba^2+^ (Figures 6B,D) when standard internal or fluoride-free internal solution was used. In fact, we observed a slightly higher success rate for available wells that passed the quality control criteria for current (I > 350 pA at 60 mV) after internal free Ca^2+^ application and R_Seal_ > 0.25 GΩ before internal free Ca^2+^ application when fluoride-free solution was used (Figure 6C), 70.3 ± 6.9% (*n* = 9 chips) vs. 60.6 ± 9.4 (*n* = 8 chips) when standard internal solution was used. Interestingly, when we repeated the same experiment using 4X NPC-384FF chips (quality check criteria for current I > 1 nA at 60 mV) the success rate increased to 94.5 ± 3.5% (*n* = 2 chips) and 91.2 ± 6.6 (*n* = 2 chips) when standard internal solution was used. This confirms the benefit of multi-hole chips for assay development in general, and as additional tool for experiments using fluoride-free internal solution.

Using increasing concentrations of internal free Ca^2+^, we could estimate the apparent affinity for internal free Ca^2+^ to elicit K_Ca_3.1 currents (Figure 6E). Both inward (measured at −120 mV) and outward currents (measured at 60 mV) were analyzed, and concentration response curves constructed for standard and fluoride-free solution at the different potentials. The concentration response curves for internal Ca^2+^ are shown overlaid in Figure 6E. The corresponding current analysis shows that when standard or fluoride-free solution was used, significantly larger peak currents were elicited at 0.3, 1, and 3 μM free internal Ca^2+^ compared with control (0 μM free internal Ca^2+^) recorded at 60 mV (*P* < 0.001), whereas only 1 and 3 μM free internal Ca^2+^ elicited a significantly larger peak amplitude compared with control (0 μM free internal Ca^2+^) recorded at −120 mV (*P* < 0.001), regardless of the internal solution used (Supplementary Figure 2). The data was normalized to the maximum response and fitted using a standard Hill equation (Figure 6E). The EC_50_ for internal Ca^2+^ was significantly lower when fluoride-free internal solution was used compared with standard internal solution at both 60 mV (635 ± 20 nM (*n* = 121) vs. 808 ± 50 nM (*n* = 66; *P* < 0.01)) and −120 mV (676 ± 22 nM (*n* = 121) in fluoride-free solution vs. 773 ± 44 nM (*n* = 66) in standard internal (*P* < 0.05)).

We also determined the IC_50_ for external Ba^2+^ using either standard internal or fluoride-free internal at −120 and 60 mV using 4X NPC-384 and 4X NPC-384FF chips, with the same ramp protocol shown in Figure 6B. Figure 6F shows the concentration response curves for Ba^2+^ at −120 and 60 mV in standard and fluoride-free solution. Ba^2+^ was added as a single concentration to each well and the concentration response curves were constructed across the whole plate where it blocked the current at −120 mV with a higher potency than at 60 mV. Figure 6F shows the average concentration response curves for 2 NPC-384 or 2 NPC-384FF chips. The IC_50_ for Ba^2+^ (mean ± S.D.) was 170 ± 8 μM when standard and 158 ± 15 μM when fluoride-free internal solution was used at a voltage of −120 mV. The IC_50_ increased ∼3-fold to 540 ± 105 μM when standard and 728 ± 161 μM at 60 mV when fluoride-free internal solution was used.

From these results we conclude that the recording of Ca^2+^-activated K-channels is possible under standard as well as fluoride-free conditions with similar success rates, and even higher success rates for available wells after Ca^2+^ activation with 4X chips than with 1X chips, making multi-hole chips a useful tool for recording Ca^2+^-activated K-channels. Given that the EC_50_ for Ca^2+^ was lower with fluoride-free internal solution, and peak currents were larger, we suggest that fluoride-free internal solution may provide an advantage over standard fluoride-containing internal solutions for recording Ca^2+^ activated channels.

## Discussion

There is no doubt that automated patch clamp has become an integral part of many electrophysiological assays involving ion channels, including safety pharmacology, lead optimization, target validation, and channelopathy research (Obergrussberger et al., 2021, 2022). Unlike manual patch clamp, the cell cannot be chosen, nor can the patch clamp aperture be moved to improve the seal. In APC, the patch clamp aperture is stationary and solely suction is used to attract the cell to the hole. In both manual and automated patch clamp, suction is then used to further improve the seal and to break into the whole cell configuration. Fluoride has been used in the internal solution in manual and automated patch clamp for years to assist with seal formation (Zeng et al., 2008; Föhr et al., 2021), in particular to record voltage-gated sodium channels where seal resistance and access parameters are critical for good voltage control. The mechanism is thought to involve CaF_2_ crystals forming at the aperture (Lojkner et al., 2019). However, it is well-documented that fluoride causes shifts in the activation and inactivation kinetics of Na_V_ channels (Meadows et al., 2002; Rugiero et al., 2003; Coste et al., 2004; Jarecki et al., 2008), as well as affecting persistent calcium current in neurons (Kay et al., 1986), activating G-proteins through interaction with Al^3+^ (Yatani and Brown, 1991; Li, 2003), and inhibits phosphatase activity (Khatra and Soderling, 1978).

### Fluoride has little effect on hERG currents

As expected, our data show that success rate as measured by % available wells using strict quality control parameters including R_Seal_ > 1 GΩ or 0.25 GΩ and I_Peak_ > 150 pA was higher when fluoride was used in the internal solution compared with fluoride-free internal solution when HEK cells stably expressing the hERG channel were used. However, with a success rate of around 60% for usable wells (R_Seal_ > 0.25 GΩ) when K-gluconate was used, this results in over 200 usable wells in each experiment lasting approximately 20 min and thus, a vast increase in throughput compared with manual patch clamp. Importantly, there was little change in the number of usable wells at the end of the experiment compared with at the start, indicating that fluoride is not absolutely necessary for long-lasting recordings and 60% for completed experiments is an acceptable success rate for screening.

When fluoride was replaced by gluconate in the internal solution, hERG-mediated currents were slightly, but statistically significantly, larger than when fluoride was present (*P* < 0.05, unpaired Student’s *t*-test). This has not been previously reported and the reasons for the difference are not clear, additional studies are required to elucidate the underlying root cause. In our experiments, we can exclude differences in cell size since the cell capacitance values were unchanged in fluoride and fluoride-free solution, and we have also shown that fluoride does not shift the V_half_ of the hERG tail current. This is in agreement with Zeng et al. (2008, 2013) who also reported no effect on tail current activation parameters by fluoride compared with chloride when using the automated patch clamp system, the PatchXpress, and manual patch clamp, although the fluoride vastly increased success rate on the PatchXpress from 10% in K-chloride to 75% in K-fluoride (Zeng et al., 2008). Indeed, we also found no difference in success rate for K-chloride-containing internal solution compared with K-gluconate-containing internal solution (Supplementary Table 3) and can conclude that both gluconate and chloride are suitable alternatives to fluoride for recordings of hERG-HEK on the SyncroPatch 384.

We also compared the IC_50_ values for two compounds, terfenadine and verapamil, obtained using K-fluoride or K-gluconate and found that the IC_50_s were not different and agreed well with the range found in the literature (Zeng et al., 2008; Kramer et al., 2013; Crumb et al., 2016; Brinkwirth et al., 2020). From our results and those of Zeng et al. (2008, 2013) we can conclude that fluoride does improve the success rate based on R_Seal_ and completed experiments and does not affect V_half_ of hERG tail current nor pharmacology, at least for the two compounds tested. Therefore, we can further conclude that the use of fluoride in the internal solution for hERG screening can be an advantage due to improved success rate, but fluoride-free internal solution can be used on the SyncroPatch 384 with success rates compatible with high throughput screening.

### Fluoride shifts V_half_ of activation and inactivation of Na_V_1.5 and Na_V_1.7

CHO cells expressing Na_V_1.5 were also used on the SyncroPatch 384 in fluoride-free internal solution with similar success rates to HEK expressing hERG indicating that this method can be applied to different cell types and different ion channels. Average R_Seal_ values were >1 GΩ regardless of internal solution used, and no difference in I_Peak_ in fluoride-free internal solution compared with standard fluoride internal solution was observed which contrasts to the results observed with hERG.

Our results show that fluoride in the internal solution causes a negative shift in the V_half_ of activation and inactivation of both Na_V_1.5 (Figures 4C,D) and Na_V_1.7 (Figure 4E) which agrees with previous reports that fluoride shifts the V_half_ of activation and inactivation to more negative values of Na_V_1.3 (Meadows et al., 2002), Na_V_1.7 (Jarecki et al., 2008), and Na_V_1.9 (Rugiero et al., 2003; Coste et al., 2004). The values for V_half_ of activation and inactivation for Na_V_1.5 and Na_V_1.7 were in good agreement with the literature for fluoride internal (Sheets and Hanck, 1999; Li et al., 2017). Importantly, despite shifts in the V_half_ of activation and inactivation, the IC_50_ of tetracaine block of Na_V_1.5 was not statistically different in fluoride-free internal solution compared with standard fluoride (Table 3 and Figures 5E,F). Changing the holding potential from −120 to −100 mV or −80 mV resulted in a more potent IC_50_ of tetracaine, regardless of fluoride or fluoride-free internal solution was used (see Supplementary Table 1) as expected, as tetracaine and other local anesthetics are state dependent blockers of sodium channels (Hille, 1977; Hondeghem and Katzung, 1977; Bean et al., 1983; Li et al., 1999). The result is somewhat surprising given the fact that internal fluoride acts in a hyperpolarizing fashion and tetracaine is dependent on the holding potential used and may point towards related but independent mechanisms of modulation during transitions between closed, open and inactivated states of Na_V_1.5 channels as suggested for Na_V_1.8 and Na_V_1.9 channels (Coste et al., 2004).

A shift in the activation and inactivation kinetics for cardiac Na_V_1.5 channels over time has been reported for canine cardiac Purkinje cells (Hanck and Sheets, 1992), rat ventricular cardiac myocytes (Maltsev and Undrovinas, 1997), and rabbit atrial myocytes (Wendt et al., 1992) using manual whole cell patch clamp. In a small study using Na_V_1.5 expressed in CHO cells, our results also indicate a negative shift in the V_half_ of activation and inactivation over time (data not shown) as previously reported (Hanck and Sheets, 1992; Wendt et al., 1992; Maltsev and Undrovinas, 1997) when activation and inactivation IVs were measured after 10 min compared with immediately after achieving the whole cell configuration. The V_half_ of activation shifted by −0.78 mV/min in fluoride internal and −0.43 mV/min in fluoride-free internal solution. The V_half_ of inactivation shifted by −1.22 mV/min in fluoride and −0.38 mV/min in fluoride-free. In this respect, although the shift in parameters appeared to be more pronounced when fluoride was present, the absence of fluoride did not completely abolish this shift in agreement with Wendt et al. (1992) who also reported a shift in the activation and inactivation parameters regardless of whether fluoride, chloride, aspartate, or glutamate were present in the internal solution. It has been proposed that the shift in activation is dependent on the cytoskeleton (Maltsev and Undrovinas, 1997) and is prevented using the perforated patch technique (Wendt et al., 1992). All our experiments were performed in the whole cell mode of the patch clamp technique, but the use of a perforator in the internal solution would allow perforated patch experiments to be performed.

Temperature has also been shown to affect activation and inactivation of Na_V_ channels where cooled temperatures cause a depolarizing shift in the V_half_ of activation of Na_V_1.3, 1.5, 1.6, and 1.7 (Kriegeskorte et al., 2022). Increasing the temperature to physiological temperature (36 ± 1°C) can also mildly shift the V_half_ of inactivation to more hyperpolarized potentials (Rotordam et al., 2021). Increased temperatures have also been shown to affect IC_50_s on hERG current, e.g., erythromycin (Kirsch et al., 2004; Stoelzle et al., 2011) and Na_V_1.5, e.g., mexiletine (Rotordam et al., 2021). For these reasons, it may be desirable to perform automated patch clamp experiments at physiological temperature. Recording at physiological temperature is more challenging and can result in a lower success rate and shorter recordings. In preliminary experiments at physiological temperature, we found that there was little change in the success rate when fluoride was used in the internal solution (see Supplementary Table 2) but the success rate in fluoride-free solution was slightly lower compared with room temperature [30.0 ± 7.1 (6 chips) for R_Seal_ > 1 GΩ at physiological temperature vs. 41.9 ± 8.6% (17 chips) at room temperature see Supplementary Table 2 and Section “High throughput APC to assess effects of internal fluoride on success rate and seal resistance”], nevertheless, and average R_Seal_ was > 1 GΩ in both conditions (Supplementary Table 2) and recordings lasted approximately 20 min (data not shown).

In conclusion for Na_V_ channels, we could confirm that fluoride shifts the V_half_ of activation and inactivation of Na_V_1.5 and Na_V_1.7 as has been reported for other Na_V_ channels (Meadows et al., 2002; Rugiero et al., 2003; Coste et al., 2004; Jarecki et al., 2008) but does not alter the IC_50_ of tetracaine on Nav1.5 channels. Success rates are higher when using fluoride in the internal solution, as expected, and many labs using both conventional and automated patch clamp use internal fluoride when recording Na_V_ channels and this must be taken into consideration when comparing kinetic parameters with the literature.

### Experiments involving Ca^2+^-activated ion channels

Intracellular Ca^2+^ activated ion channels pose a challenge to APC devices given the use of internal fluoride solutions and the very low solubility of CaF_2_ (K_sp_ = 3.5 × 10^–11^) resulting in unstable amounts of free internal Ca^2+^ concentrations. Nevertheless, the use of appropriate buffer solutions and chelating agents do allow for the development of high throughput drug discovery on Ca^2+^-activated K-Channels [please see Section “Materials and methods” (see Table 1) or Srinivasan et al. (2022)]. Our aim was to compare this approach to our recently developed fluoride-free method using a Ca^2+^-activated K-channel.

When single hole chips were used, the success rate based on seal resistance did not differ much between standard and fluoride-free solutions, which contrasts to the success rate for cells expressing hERG or Na_V_1.5, however, the success rate based on current amplitude after activation with 1 μM internal free-Ca^2+^ was increased in experiments where fluoride-free solution was used (Figures 6A,C). Additionally, current amplitudes were larger in fluoride-free internal solution (Supplementary Figure 2) and the EC_50_ for Ca^2+^ slightly lower (Figure 6E). Taken together, it would appear, for Ca^2+^-activated ion channels at least, fluoride-free internal solution should be recommended. When fluoride is present, there is most probably an unknown amount of precipitation of CaF_2_, even if this is not visible, and this can affect the apparent Ca^2+^ affinity, not to mention fluoride interfering with intracellular signaling process involving, for example, phosphorylation (Khatra and Soderling, 1978; Li, 2003). Activation of Ca^2+^-activated K-channels using fluoride is possible on high throughput APC devices, as shown recently for the BK channel by Srinivasan et al. (2022), however, the free internal Ca^2+^ may be more stable in fluoride-free conditions, especially over time/during the course of the day where precipitation cannot be completely prevented. The use of multi-hole chips with four holes per well (4X) is also possible thus improving the success rate based on current amplitude for both standard and fluoride-free internal solution to over 90%.

## Conclusion

In keeping with the topic of this special issue: “Targeting Ion Channels for Drug Discovery: Emerging Challenges for High Throughput Screening Technologies,” we have overcome the challenge of low success rates and low seal resistances on high throughput automated patch clamp devices when using fluoride-free physiological internal solution. Implementation of specialized consumables (NPC-384FF) enables us to report success rates for fluoride-free recordings of approximately 60–80% for R_Seal_ ≥ 0.25 GΩ and 40–50% for R_Seal_ ≥ 1 GΩ. We mainly used a K-gluconate-based internal solution, but we observed similar success rates using K-chloride-based internal solution (Supplementary Table 3). Recordings were also stable, typically lasting around 20 min where cumulative additions of compound or single additions of compound and incubation times of 5 min in compound were used, as per best practice considerations recommended previously for hERG and Na_V_1.5 (Brinkwirth et al., 2020; Rotordam et al., 2021). In the case of hERG, since fluoride does not appear to exert an obvious influence over inactivation parameters or IC_50_ values, the user is free to choose whether to use fluoride or fluoride-free internal solution and may prefer fluoride-containing solutions to maximize success rates and minimize cost per data point. For experiments involving Na_V_ channels, the user should be aware that fluoride causes shifts in activation and inactivation parameters and caution should be used when comparing data generated in different solutions. For recording Ca^2+^-activated channels, success rates for completed experiments are similar under both conditions, however, the response to internal free Ca^2+^ and estimation of its apparent affinity may be more favorable to pursue when fluoride-free internal solution is used due to complete absence of CaF_2_ precipitation and, therefore, there is an advantage for the user to be able to utilize fluoride-free solution with success rates exceeding 50% for completed experiments. Coupling fluoride-free internal solution with multi-hole chips increases the success rate for available wells to >90% and is, therefore, also a consideration when designing the experiment in ion channel drug screening.

**FIGURE 7:**
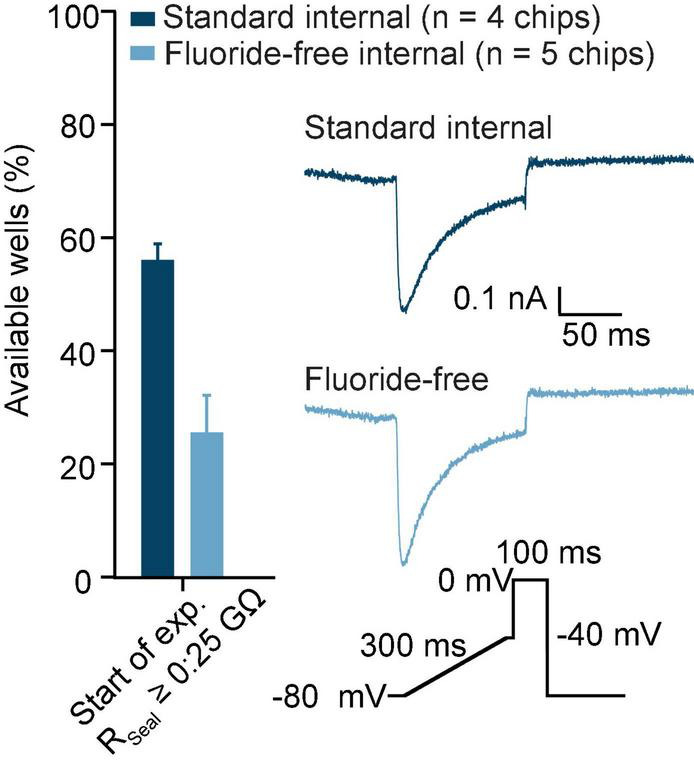
hiPSC-CMs recorded in standard and fluoride-free internal solution. hiPSC-CMs were captured with almost 60% success rate for R_Seal_ ≥ 0.25 GΩ when using standard internal solution and about 25% R_Seal_ ≥ 0.25 GΩ in fluoride-free internal solution in preliminary experiments. The inset shows an example of a Ca_V_ current recorded from an example cell in standard (top, dark blue) and fluoride-free (bottom, light blue). The voltage protocol used is also shown and only response to voltage step to 0 mV is shown in the raw trace examples.

Additionally, in preliminary recordings we could successfully use the fluoride-free approach for experiments involving stem cell-derived cardiomyocytes (Figure 7). Therefore, the approach is not limited to standard cell lines but could be adopted for a wide range of cell types and experiments to bring conditions used to generate high throughput APC data closer and more comparable to recordings done using manual patch clamp and physiological solutions. Ultimately, this will enhance ion channel characterization and compound testing when using not only cell lines, but also induced pluripotent stem cells and primary cells.

## Data availability statement

The raw data supporting the conclusions of this article will be made available by the authors, without undue reservation.

## Author contributions

MR designed the study, performed the experiments and analysis, and contributed to writing and proofreading the manuscript. AO designed the experiments, analyzed the data, made the figures, and wrote and proofreading the manuscript. SS, TG, NB, and MGR performed the experiments and contributed to writing and proofreading the manuscript. IR-W, TS, and AR performed the experiments and analysis and contributed to writing and proofreading the manuscript. SF designed the study, performed analysis, and contributed to writing and proofreading the manuscript. NF contributed to writing and proofreading the manuscript. AL, FS, and NV provided the iPSC derived cardiomyocytes, helped with the design of experiments, and contributed to writing and proofreading the manuscript. All authors contributed to the article and approved the submitted version.
