# Supplementary material for: There is no F in APC: Using physiological fluoride-free solutions for high throughput automated patch clamp experiments
Source: Front Mol Neurosci. 2022 Aug 22;15:982316. doi: 10.3389/fnmol.2022.982316 (PMC9443850; doi:10.3389/fnmol.2022.982316)
Supplement: Supplementary file 1 [file Data_sheet_1.pdf]

Supplementary Material

Supplementary Table 1: IC_{50} values for tetracaine at different holding potentials (HP) for an average of 4 chips in standard internal solution and 3 chips in fluoride-free internal solution. Single addition of compound to each well was performed and the concentration response curve calculated across multiple wells. Shown are mean ± S.E.M.

**Parameter**	**Standard internal**	**Fluoride-free internal**
IC_{50} Tetracaine HP -120 mV (µM)	35.60 ± 9.41	41.76 ± 9.29
IC_{50} Tetracaine HP -100 mV (µM)	14.76 ± 4.43	21.28 ± 5.29
IC_{50} Tetracaine HP -80 mV (µM)	5.20 ± 1.64	10.28 ± 2.49

Supplementary Table 2: Success rate, R_{Seal} and I_{Peak} parameters for CHO cells expressing $Na_V1.5$ at physiological temperature. R_{Seal} values > 4 GΩ could not be accurately calculated, therefore only $R_{Seal} \leq 4$ GΩ were used for the calculation.

**Parameter**	**Standard internal**	**Fluoride-free internal**
Success rate (% available wells) $R_{Seal} \geq 1$ GΩ at start of experiment	79.6 ± 4.7 (10 chips)	30.0 ± 7.1 (6 chips)
Success rate (% available wells) $R_{Seal} \geq 250$ MΩ at start of experiment	88.8 ± 4.5 (10 chips)	35.5 ± 9.2 (6 chips)
R_{Seal} (GΩ)	2.11 ± 0.06 (286)	1.41 ± 0.07 (156)
I_{Peak} (nA)	-2.40 ± 0.20 (357)	-1.61 ± 0.09 (194)

Supplementary Table 3: Success rate in % for the different channels/cell lines tested with different fluoride free approaches, R_{Seal} and $I_{Peak/Tail}$ cut off at R_{Seal} > 1 GΩ or R_{Seal} > 0.25 GΩ as indicated. n.d = not done

**Channel/ Cell line**	**Parameter**	**Fluoride-free internal (K-Gluconate based)**	**Fluoride-free internal (K-Chloride based)**
Nav1.5-CHO	Success rate (% available wells) $R_{Seal} \geq 1$ GΩ & I < -100 pA	Start: 41.9 ± 9.3 (17 chips)<br><br>End: 33.0 ± 5.7 (17 chips)	Start: 37.8 ± 6.6 (6 chips)<br><br>End: 30.2 ± 4.4 (6 chips)
Nav1.5-CHO	Success rate (% available wells) $R_{Seal} \geq 250$ MΩ & I < -100 pA	Start: 55.0 ± 11.7 (17 chips)<br><br>End: 46.3 ± 8.6 (17 chips)	Start: 57.4 ± 5.8 (6 chips)<br><br>End: 48.9 ± 6.1 (6 chips)
hERG-CHO	Success rate (% available wells) $R_{Seal} \geq 1$ GΩ & I > 150 pA	n.d	Start: 38.0 ± 7.1 (2 chips)<br><br>End: 32.0 ± 8.5 (2 chips)
hERG-CHO	Success rate (% available wells) $R_{Seal} \geq 250$ MΩ & I > 150 pA	n.d	Start: 63.5 ± 9.2 (2 chips)<br><br>End: 57.5 ± 6.4 (2 chips)
hERG-HEK	Success rate (% available wells) $R_{Seal} \geq 1$ GΩ & I > 150 pA	Start: 35.9 ± 7.9 (32 chips)<br><br>End: 33.6 ± 7.8 (32 chips)	Start: 36.9 ± 8.3 (2 chips)<br><br>End: 32.2 ± 6.4 (2 chips)
hERG-HEK	Success rate (% available wells) $R_{Seal} \geq 250$ MΩ & I > 150 pA	Start: 59.0 ± 8.5 (32 chips)<br><br>End: 57.0 ± 8.8 (32 chips)	Start: 47.8 ± 3.8 (2 chips)<br><br>End: 45.3 ± 7.0 (2 chips)

1 Supplementary Figures and Tables

For more information on Supplementary Material and for details on the different file types accepted, please see here. Figures, tables, and images will be published under a Creative Commons CC-BY licence and permission must be obtained for use of copyrighted material from other sources (including re-published/adapted/modified/partial figures and images from the internet). It is the responsibility of the authors to acquire the licenses, to follow any citation instructions requested by third-party rights holders, and cover any supplementary charges.

1.1 Supplementary Figures

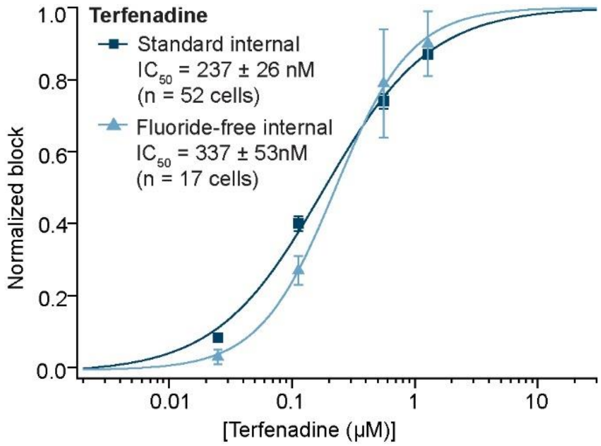

Supplemental Figure S1: Concentration response curve for Terfenadine in standard internal solution or fluoride-free solution are shown overlaid.

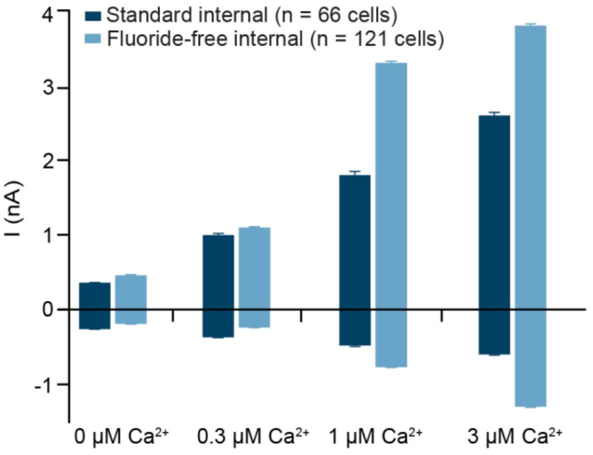

Supplementary Figure S2. Mean I_{Peak} values observed after applying 0, 0.3, 1 and 3 µM free internal Ca^{2+} in standard internal solution or fluoride-free solution at -120 mV (bars below 0 nA) or 60 mV (bars above 0 nA).
